# Evolution of ceftazidime-avibactam resistance driven by variation in *bla*_KPC-2_ to *bla*_KPC-190_ during treatment of ST11-K64 hypervirulent *Klebsiella pneumoniae*

**DOI:** 10.3389/fcimb.2025.1607127

**Published:** 2025-06-06

**Authors:** Zeshi Liu, Jing Lei, Xue Zhang, Jian Yin, Yanping Zhang, Ke Lei, Yan Geng, Lingjuan Huang, Qiang Han, Aili He

**Affiliations:** ^1^ Department of Clinical Laboratory, The Second Affiliated Hospital of Xi'an Jiaotong University, Xi'an, China; ^2^ Geriatrics Department, The First Affiliated Hospital of Xi'an Medical College, Xi'an, China; ^3^ Department of Gynaecology and Obstetrics, The Second Affiliated Hospital of Xi'an Jiaotong University, Xi'an, China; ^4^ Department of Hematology, The Second Affiliated Hospital of Xi'an Jiaotong University, Xi'an, China; ^5^ National-Local Joint Engineering Research Center of Biodiagnostics and Biotherapy, The Second Affiliated Hospital of Xi'an Jiaotong University, Xi'an, China; ^6^ Xi’an Key Laboratory of Hematological Diseases, The Second Affiliated Hospital of Xi’an Jiaotong University, Xi'an, China

**Keywords:** KPC-190, *Klebsiella pneumoniae*, ceftazidime-avibactam, blaKPC variants, antibiotic resistance

## Abstract

**Introduction:**

The emergence of *Klebsiella* pneumoniae carbapenemase (KPC) variants has significantly compromised the efficacy of ceftazidime-avibactam (CZA), a critical antibiotic for treating carbapenem-resistant *K. pneumoniae* (CRKP) infections. This study investigates the novel KPC-190 variant, identified in a hypervirulent ST11-K64 *K. pneumoniae* strain during CZA therapy, which confers resistance to CZA while partially restoring carbapenem susceptibility.

**Methods:**

The *K. pneumoniae* clinical isolate LX02 harboring *bla*_KPC-190_ was characterized using antimicrobial susceptibility testing, whole-genome sequencing (Illumina and Nanopore), and plasmid analysis. Functional studies included plasmid transformation, cloning assays, and enzyme kinetics (spectrophotometric analysis of purified KPC-190 protein). Genetic context was mapped using bioinformatics tools (RAST, ResFinder, Proksee), and virulence determinants were identified.

**Results:**

KPC-190 exhibited a unique resistance profile: high-level CZA resistance (MIC >64 μg/mL) with reduced carbapenem MICs (imipenem MIC = 2 μg/mL). Enzyme kinetics revealed decreased *K*cat/Km for carbapenems and ceftazidime, alongside a 9-fold higher IC50 for avibactam (0.13 μM vs. KPC-2’s 0.014 μM). Genomic analysis identified *bla*_KPC-190_ within an IS26 flanked mobile element (IS26-ISKpn8-*bla*_KPC_-ΔISKpn6-ΔtnpR-IS26) on an IncFII plasmid. The strain also carried hypervirulence markers (*rmpA2*, *iucABCD-iutA*, and type 1/3 fimbriae).

**Discussion:**

The KPC-190 variant underscores the adaptive evolution of *bla*_KPC_ under antibiotic pressure, combining CZA resistance via enhanced ceftazidime affinity and avibactam evasion with retained carbapenem hydrolysis. Its association with hypervirulence plasmids and IS26-mediated mobility poses a dual threat for dissemination. These findings highlight the urgent need for genomic surveillance and alternative therapies (e.g., meropenem-vaborbactam) to address KPC-190-mediated resistance.

## Introduction

Carbapenem-resistant *Klebsiella pneumoniae* (CRKP) poses a severe threat to global healthcare systems due to its ability to evade the effects of multiple antibiotics (Gonçalves Barbosa et al., 2022; Chou et al., 2024). A major driver of carbapenem resistance in CRKP is the production of *Klebsiella pneumoniae* carbapenemases (KPCs), encoded by the *bla*_KPC_ gene. Ceftazidime-avibactam (CZA), a combination of a third-generation cephalosporin and an innovative β-lactamase inhibitor, was designed to effectively target carbapenemase-producing pathogens, including strains that produce Ambler class A, class C and some class D β-lactamases (Sharma et al., 2016; van Duin and Bonomo, 2016). Studies have shown the superiority of ceftazidime-avibactam over colistin in the initial treatment of the infections caused by CRE (carbapenem-resistant Enterobacterales), and the use of ceftazidime-avibactam can reduce hospital mortality from all causes and improve risk-benefit outcomes (Karaiskos et al., 2021; Castón et al., 2022). However, since its introduction in China in 2019, ceftazidime-avibactam has exerted selective pressure on KPC-producing K. pneumoniae populations, particularly in nosocomial settings (Boattini et al., 2024). While metallo-β-lactamase-producing strains exhibit intrinsic resistance, recent studies demonstrate the emergence of ceftazidime-avibactam-resistant variants specifically among KPC-producers through acquired mutations (Oliva et al., 2024).

The resistance mechanisms of ceftazidime-avibactam predominantly involve the alterations of the major porins that allow the diffusion of CZA across the outer membrane (*OmpK35* and *OmpK36* porins), the increased gene expression and/or copy number of *bla*_KPC_, and alterations in cell permeability or efflux pump activity, with amino acid substitutions in the KPC enzyme constituting a primary mechanism (Wang et al., 2020). Since its discovery, more than 200 *bla*_KPC_ variants have been reported globally, with variations in *bla*_KPC-2_ being the primary mechanism for the evolution of these variants. Amino acid variations in the *bla*_KPC_ gene are primarily located in the loop region near the active site (Hobson et al., 2020). These variations often result in resistance to advanced β-lactam/β-lactamase inhibitor combinations, such as ceftazidime-avibactam. Notably, these resistant isolates may demonstrate cross-resistance to FDC (cefiderocol), which has been attributed to multiple mechanisms including structural similarities between FDC and ceftazidime, porin modifications, and enhanced efflux pump activity (Findlay et al., 2025). The mechanism underlying the resistance to FDC is a combination of different mechanisms including the co-expression of different β-lactamases (NDM (New Delhi metallo-β-lactamase) and KPC variants), variations affecting siderophore–drug receptor expression/function (*cir*A and *fiu* of *Escherichia coli*), overexpression of efflux pumps (*OmpK3*5 and *OmpK36*), and target modification (penicillin-binding protein 3, PBP-3 coded by the *fts*I gene) (Karakonstantis et al., 2022; Lan et al., 2022).

This study describes the first identification of KPC-190 in a clinical setting, isolated from a patient undergoing prolonged ceftazidime-avibactam therapy. Through whole-genome sequencing and enzymatic characterization, we explored the genetic and functional features of KPC-190, shedding light on its resistance profile and implications for therapy. The findings emphasize the need for vigilant monitoring and innovative treatment strategies for combating KPC-190-mediated resistance.

## Methods

### Strain identification and antimicrobial susceptibility testing

Strain identification was performed using MALDI-TOF MS (bioMérieux, France). Minimal inhibitory concentrations (MICs) were determined by broth microdilution following Clinical and Laboratory Standards Institute (CLSI) guidelines, with *Escherichia coli* ATCC 25922 as the quality control strain. MIC interpretation adhered to CLSI breakpoints, except for tigecycline (S ≤ 0.5 mg/L; R > 0.5 mg/L) and eravacycline (S ≤ 0.5 mg/L), which followed EUCAST and FDA breakpoints, respectively. Rapid carbapenemase detection utilized a colloidal gold immunoassay (CGI) kit (Gold Mountain River Tech Development Co., Beijing, China) and the carbapenemase inhibitor enhancement test.

### Molecular characterization of carbapenemase genes

Whole-genome DNA from the isolates carrying *bla*_KPC-2_, and *bla*_KPC-190_ was extracted using the QIAamp DNA minikit (Qiagen, France). PCR amplification of *bla*_KPC_ variants employed primers Kpc-rbs (5’-CTCCACCTTCAAACAAGGAAT-3’) and Kpc-rev (5’-ATCTGCAGAATTCGCCCTTCGCCATCGTCAGTGCTCTAC-3’), and the resulting products were cloned into plasmid pHSG398 downstream of the *pLac* promoter for phenotypic studies. Recombinant plasmids were electroporated into *E. coli* DH5α and *K. pneumonia* 13882, and constructs were sequenced using T7 promoter/terminator primers. Gene sequences were analyzed via NCBI tools.

### Whole-genome sequencing and bioinformatics analysis

Genomic DNA was sequenced on the Illumina MiSeq platform (paired-end, 2×300 bp) and supplemented with Oxford Nanopore long-read sequencing. Hybrid *de novo* assembly was conducted using Unicycler v0.4.8. Resistance genes, plasmid replicons, and sequence types were annotated using RAST v2.0, MLST, PlasmidFinder, and ResFinder. Single nucleotide polymorphism (SNP) analysis was performed with Snippy v4.4.3 using *K. pneumoniae* 5055 as a reference. The genetic environment of *bla*_KPC_ was examined with VRprofile, and plasmid drafts were generated via Proksee.

### Plasmid transformation and conjugation experiments

To evaluate the transferability of ceftazidime-avibactam resistance, a conjugation experiment was performed using *Klebsiella pneumoniae* LX02 as the donor strain and *Escherichia coli* J53 and EC600 as the recipient. Transconjugants were selected on Luria–Bertani (LB) agar plates containing ceftazidime-avibactam (1 μg/mL) and sodium azide or Rifampin (150 μg/mL). In addition, a transformation experiment was carried out by extracting plasmid DNA from *K. pneumoniae* LX02 using the phenol–chloroform method, followed by electroporation into *E. coli* DH5α as the recipient strain. Transformants were grown on LB agar plates supplemented with ampicillin (50 μg/mL), screened for the presence of the *bla*_KPC_ gene via PCR, and further analyzed through sequencing and comparison using the BLAST program (http://blast.ncbi.nlm.nih.gov/Blast.cgi) (Poirel et al., 2011).

### Protein purification and enzyme kinetics

The *bla*_KPC_ coding region (amino acids 25-293) was inserted into the pET-28a expression plasmid to generate an N-terminally His-tagged recombinant protein. Following transformation into *E. coli* BL21(DE3) cells, the expressed protein was isolated using immobilized metal affinity chromatography followed by size-exclusion purification (Pemberton et al., 2017). Protein quantification was achieved through UV absorbance at 280 nm, applying a molar extinction coefficient of 39,545 M^-1^cm^-1^, with final concentrations adjusted to 10-20 mg/mL.

Enzyme activity measurements were conducted spectrophotometrically under ambient conditions using purified protein preparations. Reactions were carried out in phosphate-buffered saline (pH7.4), with kinetic constants derived by nonlinear regression analysis of the Michaelis-Menten model (Winkler et al., 2015). Nitrocefin hydrolysis was assessed by tracking absorbance changes at 482 nm across a concentration gradient. Ceftazidime turnover rates were quantified by monitoring absorbance at 257 nm, while meropenem and imipenem degradation were both followed at 299 nm, both using identical experimental setups with appropriate substrate concentration ranges.

Inhibitor potency (IC_50_) against KPC-2 and KPC-190 variants was evaluated using nitrocefin hydrolysis as reporter activity. Protein samples were pre-incubated with serial dilutions (0-30 μM) of avibactam for 10 minutes prior to substrate addition (100 μM final nitrocefin concentration). After 30 minutes incubation, 482 nm absorbance measurements were collected and dose-response curves generated using Prism analysis software.

## Results

### Overview of the *K. pneumoniae* clinical isolate

*K. pneumoniae* LX01 and LX02 were sequentially isolated from a 65-year-old male patient hospitalized at the Second Affiliated Hospital of Xi’an Jiaotong University for hospital-acquired pneumonia (HAP) complicating acute liver failure. The patient had significant comorbidities including hypertension, and type 2 diabetes.

On admission, he was empirically treated with meropenem (1 g q8h for 5 d) for suspected Gram-negative infection. Despite therapy, persistent fever (38.5-39.2°C), leukocytosis (WBC 15.3×10^9^/L), and elevated procalcitonin (2.8 ng/mL) were observed, with *K. pneumoniae* LX01 (carbapenem-resistant) isolated from sputum cultures (≥10^5^ CFU/mL). Treatment was escalated to ceftazidime-avibactam (2.5 g q8h).

**Figure 1:**
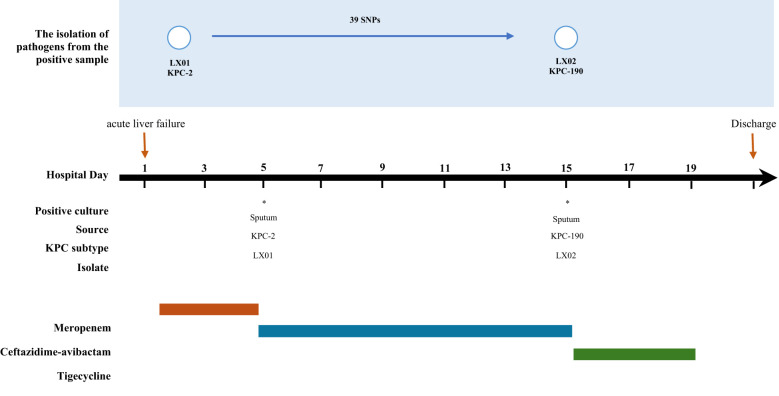
Timeline clinical history of the case patient. * indicates the isolation of KPC-positive Klebsiella pneumoniae.

After 10 days, while fever subsided, inflammatory markers remained elevated (CRP 48 mg/L, PCT 1.2 ng/mL), and *K. pneumoniae* LX02 (ceftazidime-avibactam-resistant) was isolated. Rectal screening confirmed did not carry KPC-producing *K. pneumoniae* in the intestine before treatment. Final clearance was achieved with tigecycline (100 mg q12h for 4 d), with normalization of clinical (afebrile) and laboratory (WBC 8.7×10^9^/L, CRP <10 mg/L) parameters before discharge. A comprehensive timeline of the patient’s medication history is shown in **Figure 1**.

### Antimicrobial susceptibility testing and *bla*_KPC_ gene expression

Antimicrobial susceptibility testing revealed distinct resistance profiles for the *K. pneumoniae* clinical isolates carrying KPC-2 and its variants (**Table 1**). The initial strain, LX01 (KPC-2), displayed high resistance to carbapenems (imipenem MIC = 32 μg/mL, meropenem MIC = 16 μg/mL). After 10 days of ceftazidime-avibactam therapy, the second isolate, LX02 (KPC-190), exhibited resistance to ceftazidime-avibactam (MIC > 64 μg/mL) and carbapenems (imipenem MIC = 2 μg/mL, meropenem MIC = 4 μg/mL). These findings suggest that the evolution from KPC-2 to KPC-190 resulted in partial restoration of carbapenem susceptibility, while maintaining high level resistance to ceftazidime-avibactam. The two isolates were resistant to all the β-lactams tested, quinolones, and aminoglycosides but they were susceptible to colistin and tigecycline. Concerning the BLICs, the isolate was resistant to CZA and susceptible to meropenem-vaborbactam, imipenem-relebactam and aztreonam-avibactam. Resistance to FDC (MIC = 8 mg/L) was also detected. In addition, there was a significant upward trend in the *bla*_KPC_ gene expression during treatment in *K. pneumoniae* LX02. This strain showed a 4-fold increase in gene expression when compared to the initially isolated *K. pneumoniae* LX01.

**Table 1:** Antimicrobial susceptibility profiles of clinical strains of *K. pneumoniae* (LX01 and LX02), *bla*_KPC-_positive conjugants and transformants, recipient strain.

Antimicrobials	MIC							
	LX01 (KPC-2)	LX02 (KPC-190)	pHSG398-*E. coli* DH5a	KPC-2-*E. coli* DH5a	KPC-190-*E. coli* DH5a D179Y and A243V	*K. pneumoniae* 13882-pHSG398	*K. pneumoniae* 13882-KPC-2	*K. pneumoniae* 13882-KPC-190
Imipenem	32	2	0.125	16	0.5	0.125	16	0.5
Meropenem	16	4	≤0.06	4	0.25	≤0.06	16	0.25
Meropenem-vaborbactam	2	2	≤0.06	≤0.06	≤0.06	≤0.06	≤0.06	≤0.06
Ceftazidime	>32	>32	0.5	16	128	0.5	8	128
Ceftazidime-avibactam	8	>64	0.125	0.125	32	0.125	0.5	128
Imipenem-relebactam	0.5	0.5	0.125	0.125	0.125	0.125	0.125	0.125
Amikacin	>128	>128	1	0.5	1	1	1	1
Aztreonam	>128	>128	0.125	128	1	0.125	64	32
Aztreonam-avibactam	1	2	0.125	0.125	0.125	0.125	0.125	0.125
Cefiderocol	8	8	0.06	0.06	0.06	0.06	0.06	0.06
Ciprofloxacin	>8	>8	≤0.06	≤.06	≤.06	≤0.06	≤0.06	≤0.06
Polymyxin B	0.25	0.25	0.5	0.5	0.5	0.5	0.5	0.5
Tigecycline	0.5	0.25	0.25	0.25	0.25	0.25	0.25	0.25

Grey shading indicates resistance to antimicrobials.

### WGS analysis of the *bla*_KPC_-carrying plasmid

**Figure 2:**
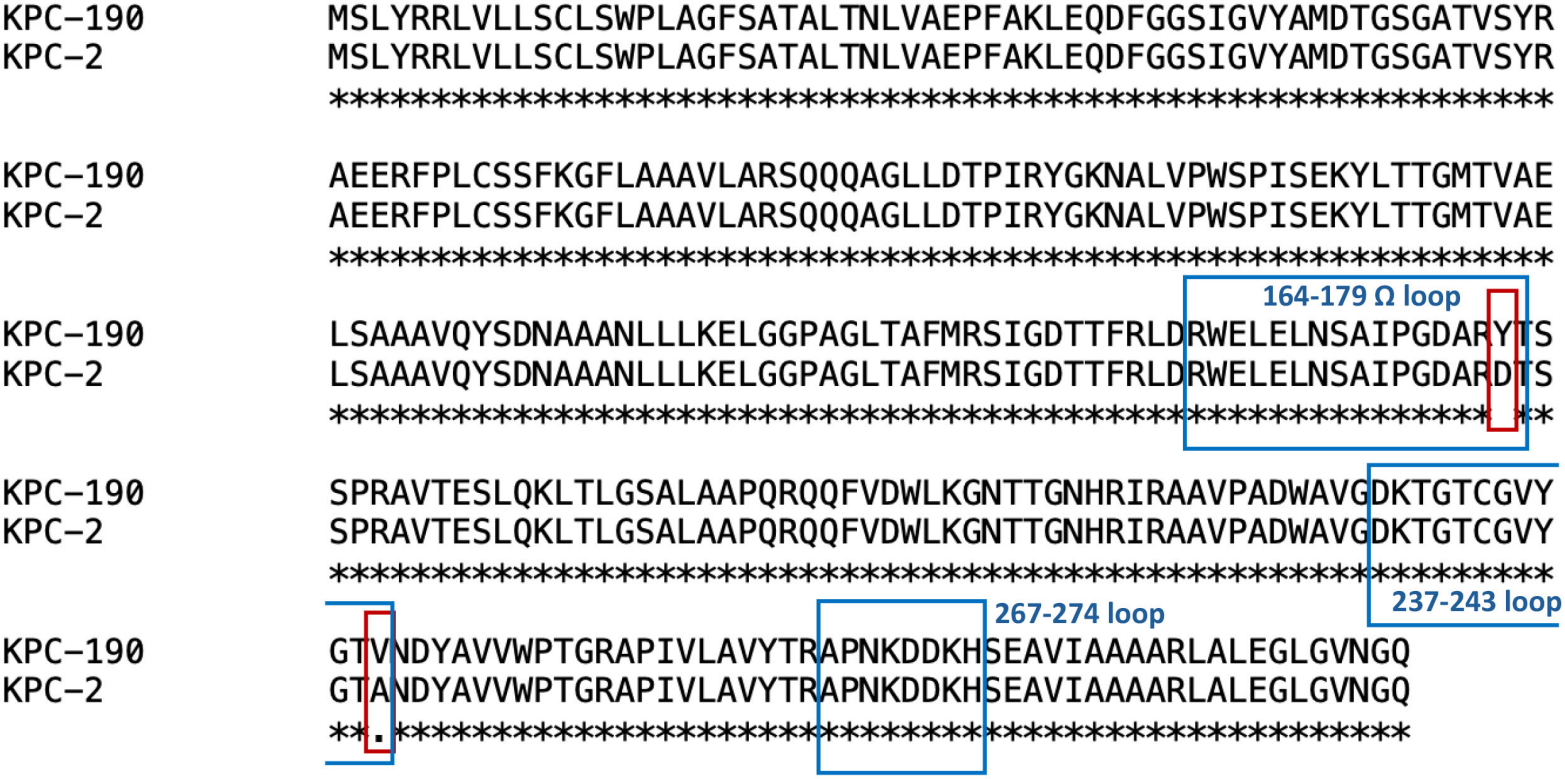
Amino acid sequence alignment of KPC-2 and KPC-190. Amino acids that don't match the reference are marked with red frame.

Comparative analysis revealed that the two isolates differed by 39 SNPs, which showed a high degree of homology (**Figure 1**). Whole-genome sequencing analysis confirmed that the two strains belonged to clone ST11-KL64 with the *iucABCD-iutA* virulence cluster and *rmpA/rmpA2* genes, which could be identified as hypervirulent *K. pneumoniae (* Li et al., 2018) in association with the patient’s poor prognosis. In addition, several resistance genes were found, including the β-lactamase genes *bla*_CTX-M-65_, *bla*_SHV-12_, and *bla*_TEM-1B_, the aminoglycoside resistance genes *rmtB* and *aadA2*, the fluoroquinolone resistance gene *qnrS1*, the fosfomycin resistance gene *fosA3*, the trimethoprim-sulfamethoxazole resistance genes *dfrA14* and *sul2*. The *bla*_KPC-2_ gene was detected in *K. pneumoniae* LX01. Notably, whole-genome comparative analysis also revealed one novel *bla*_KPC-2_ variants in *K. pneumoniae* LX02, designated *bla*_KPC-190_. Nucleotide alignment of *bla*_KPC_ variants revealed variations in both the Ω-loop and the 237-243 loop (D179Y and A243V) (**Figure 2**).

**Figure 3:**
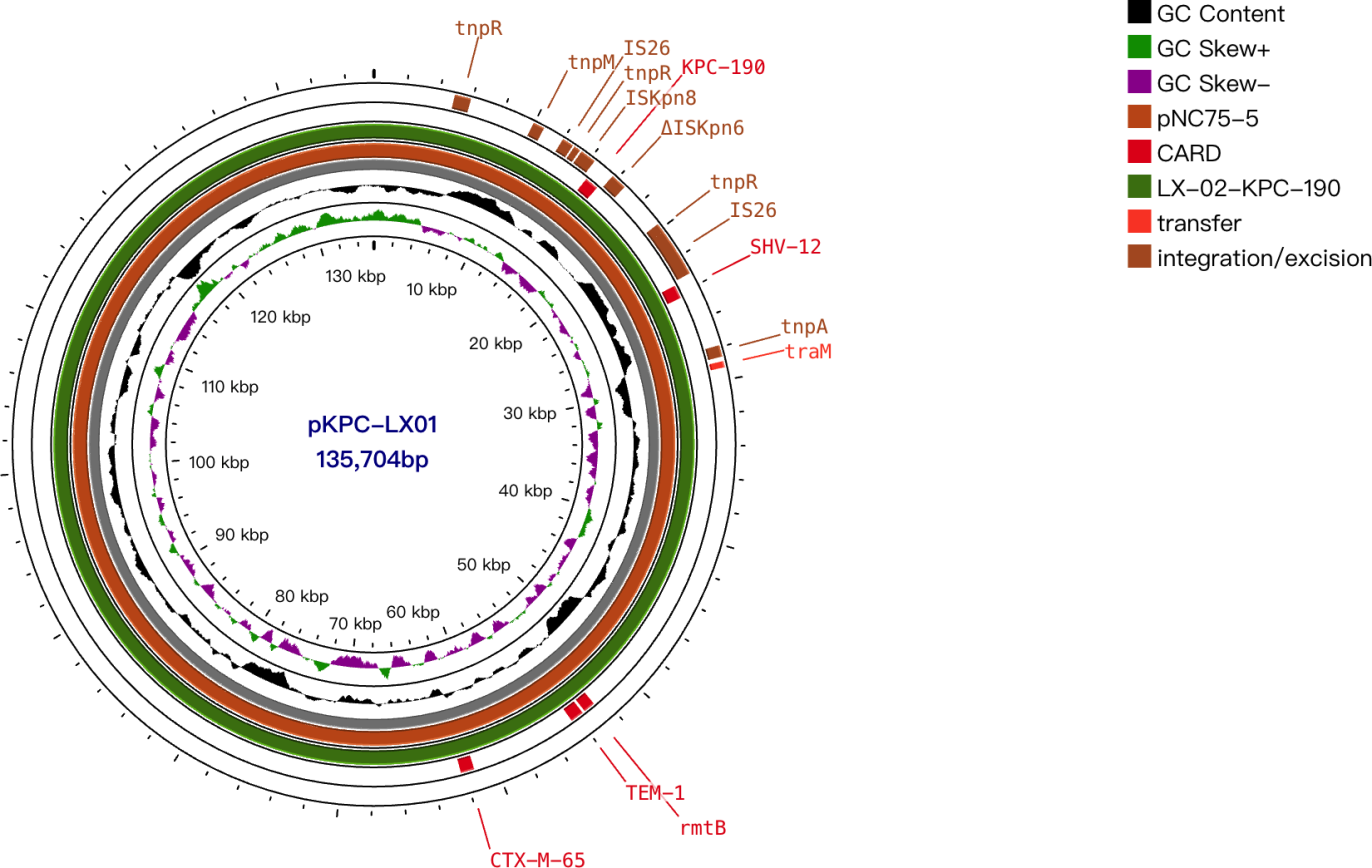
Alignments of plasmids. Comparison of the plasmids pKPC-LX01 with plasmid pNC75-5 (134,876 bp, GenBank accession no. CP163294.1). Pivotal genes related to replication, transfer, transposition, and resistance are labeled.

The complete sequence was also obtained for plasmid characterization. *bla*_KPC-2_-positive *K. pneumoniae* LX01 was used as a representative strain. This strain had five plasmids pVir-LX01, pKPC-LX01, p3-LX01, p4-LX01, and p5-LX01 with sizes of approximately 193, 136, 87, 12, 0.5kb. Virulence factors including iroN, rmpA, iucA, iucB, iucC, iucD, iutA, rmpA2 were identified on the virulence plasmid pVir-LX01. The *bla*_KPC_ was located on the IncFII-type plasmid, pKPC-LX01, which was reported to be one of the key vectors mediating transmission of *bla*_KPC_ (Yang et al., 2021). The multi-replicon enhanced the ability to replicate in a wider range of hosts (Li et al., 2019). *bla*_CTX-M-65_, *bla*_TEM-1B_, *rmtB* were also identified in this plasmid. In addition, several resistance genes including qnrS1, dfrA14 were carried by the plasmid p3-LX01. BLAST analysis revealed that the plasmid pKPC-LX01 had high coverage with the plasmid pNC75-5 in the NCBI database, which carries *bla*_KPC-2_ isolated from clinical *K. pneumoniae* (**Figure 3**).

### Plasmid transformation assay

Plasmid transformation assays yielded significant results (**Table 1**). Transformants KPC-190-*E. coli* DH5a increased the MICs of ceftazidime-avibactam and ceftazidime by at least 256- and 256-fold, respectively compared with the recipient *E. coli* DH5α. KPC-190 mediated a 4-fold increase in MICs for imipenem, for meropenem, KPC-190 mediated an at least 4-fold increase in the MIC of imipenem. Notably, no *bla*_KPC-_containing conjugants were detectable when either *E. coli* EC600 or J53 were used as recipients. The *bla*_KPC_-containing plasmids in this study do not appear to be self-conjugative, potentially due to the absence of relaxase and T4CP.

### Functional characterization of KPC variants

The recombinant plasmids carrying *bla*_KPC_ were successfully introduced into the recipient strain *K. pneumoniae* ATCC 13882. *bla*_KPC-2_ conferred resistance to most β-lactams including ceftazidime, cefepime, imipenem, and meropenem, but retained susceptibility to ceftazidime-avibactam (**Table 1**). In contrast to the *bla*_KPC-2_-positive clonal strains, the MICs of the *bla*_KPC-190_ strains decreased at least 32-fold towards carbapenems and increased at least 64-fold to ceftazidime-avibactam. In general, the *bla*_KPC-2_ variants conferred resistance to ceftazidime-avibactam and susceptibility to carbapenems compared to *bla*_KPC-2_. Importantly, the *bla*_KPC-2_ variants appeared to confer a higher ceftazidime-avibactam MIC in *K. pneumoniae* than in *E. coli.* This suggests that variations in strain backgrounds may influence the expression of *bla*_KPC_ variants. In addition, emerging carbapenemase inhibitor combinations, namely imipenem-relebactam, aztreonam-avibactam, and meropenem-vaborbactam, showed superior inhibitory performance against both *bla*_KPC-2_ and variants.

### Enzyme kinetic data

The enzyme kinetics and IC_50_ values of KPC-2 and KPC variants were determined. Enzymatic assays demonstrated that the hydrolytic activity (*Kcat/Km*) of KPC-190 against carbapenems was reduced compared to KPC-2. Hydrolytic activity against ceftazidime was significantly decreased for KPC-190 compared to KPC-2, indicating that the KPC-190 enzymes exhibited reduced hydrolysis and higher affinity for ceftazidime compared with wild-type KPC-2 (**Table 2**). Notably, the KPC variants had almost no detectable hydrolysis activity of carbapenems (ertapenem, imipenem, and meropenem), indicating reduced activity to carbapenems. To evaluate the inhibitory activity of the inhibitor against the KPC-2 and KPC-190 enzymes, the 50% inhibitory concentration (IC_50_) values for avibactam were also measured. The IC_50_ values for avibactam were markedly higher for KPC-190 (0.13 μM) compared to KPC-2 (0.014 μM), indicating a significant reduction in avibactam binding affinity. This low affinity for avibactam, coupled with the retained hydrolytic activity against carbapenems, accounts for the resistance phenotype of KPC-190.

**Table 2:** Kinetic parameters and IC_50_ of the β-lactamases KPC-2 and KPC-190.

Antimicrobials	*bla*_KPC_ variants	*K_m_* (μM)	*K_cat_* (s^-1^)	*K_cat_/K_m_* (μM^-1^s^-1^)	IC_50_(μM)
Imipenem	*bla*_KPC-2_	450.3	74	0.16	/
	*bla*_KPC-190_	522.13	62.15	0.12	/
Meropenem	*bla*_KPC-2_	96.35	89.21	0.93	/
	*bla*_KPC-190_	241.61	62.31	0.26	/
Ceftazidime	*bla*_KPC-2_	78.32	2.5	0.032	/
	*bla*_KPC-190_	87.86	0.87	0.001	/
Avibactam	*bla*_KPC-2_	/	/	/	0.014
	*bla*_KPC-190_	/	/	/	0.13

### Genetic context of *bla*_KPC-190_ and virulence factors of *K. pneumoniae* LX01

**Figure 4:**
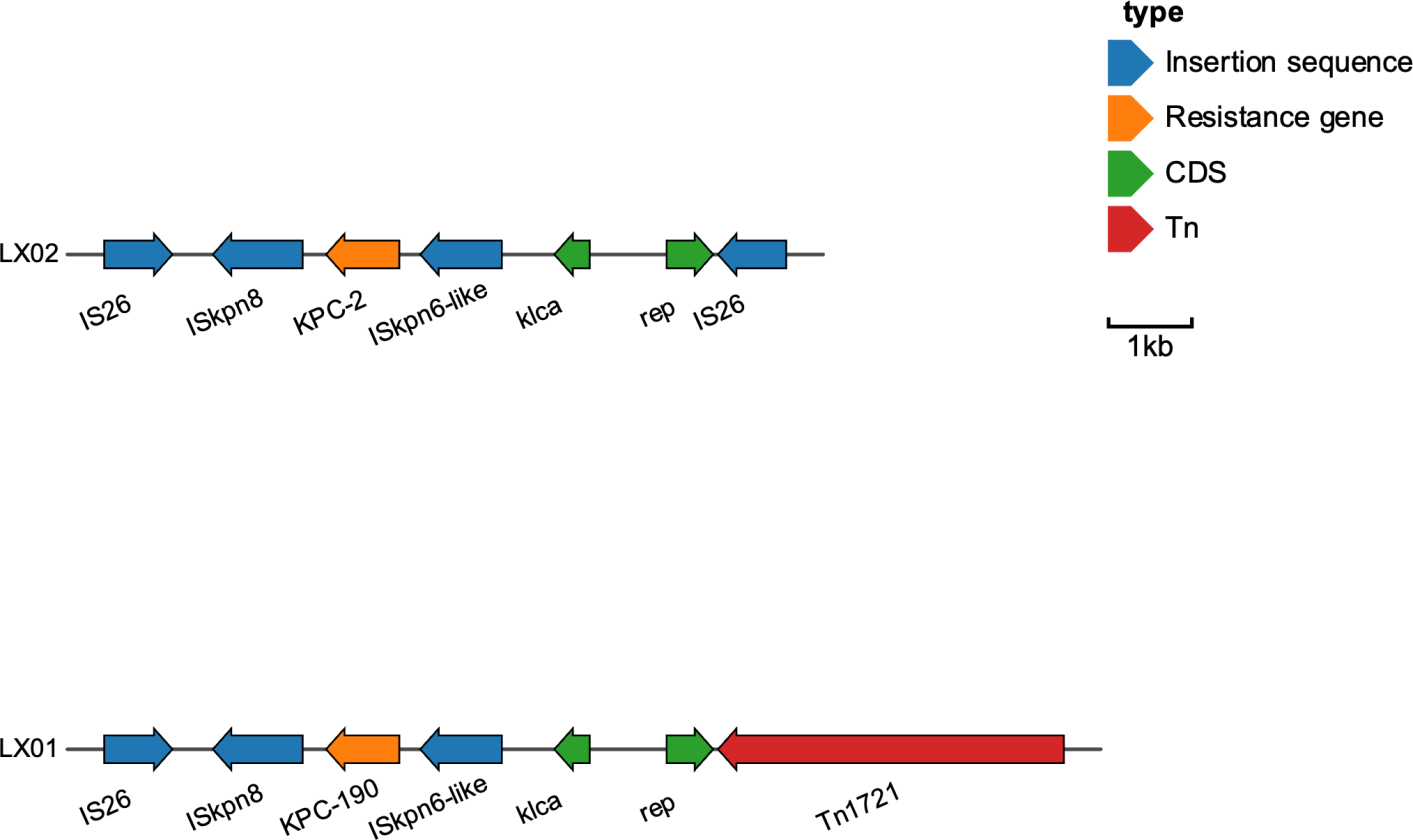
Genetic environment of blaKPC-2 and blaKPC-190 and comparative analysis.

Whole-genome sequencing revealed that *bla*_KPC-190_ is located on an IncFII plasmid within the translocatable unit: IS*26*-IS*Kpn8*-*bla*_KPC_-ΔIS*Kpn6*-ΔtnpR-IS*26*, which contrasts with previous studies where three different variants of Tn*1721* harbouring *bla*_KPC-2_ (referred to as A1-, A2-, and B-type) predominated in China (**Figure 4**). Here, IS*26* replaced Tn*1721* to form a transposable structure with double IS*26* flanking the IS*Kpn6*-*bla*_KPC_-IS*Kpn27* core structure, a configuration rarely observed in *bla*_KPC_ variants.

The ST11-K64 *Klebsiella pneumoniae* LX02 strain harbored a variety of virulence factors (**Table 3**). Notably, it contained a large virulence plasmid approximately 200 kb in size, which carried genes conferring resistance to heavy metals such as copper and silver, alongside virulence-related genes responsible for capsular polysaccharide synthesis regulation (*rmpA/rmpA2*) and iron acquisition systems (*iucABCD-iutA*). This plasmid shared significant similarity with the widely disseminated pLVPK-like plasmids (reference sequence GenBank accession number CP094941) but lacked a ∼25-kb pathogenicity island that includes *rmpA*, *fecAIR*, and *iroBCDN*.

**Table 3:** Genetic characterization of strains *K. pneumoniae* LX01 and *K. pneumoniae* LX02.

Strain	contig	Size (bp)	Resistance and virulence genes	Inc type
LX01	chromosome	5435629	*aadA2b, bla*_SHV-182_, fosA6, sul1, *yagV/ecpE, yagW/ecpD, yagX/ecpC, yagY/ecpB, yagZ/ecpA, ykgK/ecpR*, *galF, cpsACP, wzi, wza, wcaJ, gnd, manC, manB, wbtL, ugd, wzm, wzt, wbbM, glf, wbbN, wbbO, rcsA*	–
	pVir-LX01	192901	*iroN, rmpA, iucA, iucB, iucC, iucD, iutA, rmpA2*	IncHI1B, repB
	pKPC-LX01	135704	*bla*_SHV-12_ *, bla*_CTX-M-65_ *, bla*_TEM-1B_ *, rmtB, bla*_KPC-2_	IncFII
	p3-LX01	87095	*qnrS1, bla*_LAP-2_ *, tet(A), sul2, dfrA14*, catA2	–
	p4-LX01	11970	*-*	ColRNAI
	p5-LX01	5596		–
LX02	chromosome	5435629	*aadA2b, bla*_SHV-182_, fosA6, sul1, *yagV/ecpE, yagW/ecpD, yagX/ecpC, yagY/ecpB, yagZ/ecpA, ykgK/ecpR*, *galF, cpsACP, wzi, wza, wcaJ, gnd, manC, manB, wbtL, ugd, wzm, wzt, wbbM, glf, wbbN, wbbO, rcsA*	–
	pVir-LX01	192957	*iroN, rmpA, iucA, iucB, iucC, iucD, iutA, rmpA2*	IncHI1B, repB
	pKPC-LX01	135704	*bla*_SHV-12_ *, bla*_CTX-M-65_ *, bla*_TEM-1B_ *, rmtB, bla*_KPC-190_	IncFII
	p3-LX01	87095	*qnrS1, bla*_LAP-2_ *, tet(A), sul2, dfrA14*, catA2	–
	p4-LX01	11970	*-*	ColRNAI
	p5-LX01	5596		–

In addition to the plasmid, the bacterial chromosome encoded multiple virulence-associated gene clusters. These included the *fim* and *mrk* clusters, which are responsible for the production of type 1 and type 3 fimbriae, respectively. These fimbriae, in combination with capsule production, represented the primary virulence mechanisms of *K. pneumoniae* LX02. Interestingly, copy number variations (CNVs) were observed in some chromosomal genes, with a slight reduction in *mrkA* and an increase in *pgaA*.

## Discussion

The *bla*_KPC-2_-positive ST11 type CRKP strains have emerged as a formidable clonal lineage in China, posing a threat in clinical settings (Shi et al., 2020; Huang et al., 2023). Ceftazidime-avibactam, an effective antibiotic, has been clinically approved in China since 2019, with a predicted increase in resistance following extensive use (Dietl et al., 2020). Resistance to ceftazidime-avibactam in CRKP strains has typically been correlated with various variations in the *bla*_KPC-2_ gene (Wang et al., 2020). More than 60% of novel KPC gene subtypes have emerged since 2019, with variations primarily occurring in four loops surrounding the active site core: the 105-loop (Leu102 to Ser106), Ω-loop (Arg164 to Asp179), 240-loop (Cys238 to Thr243) and 270-loop (Ala267 to Ser275) (Hobson et al., 2020). Amino acid substitutions in the Ω-loop of the KPC-2, particularly at position 179, are the major contributors to resistance against ceftazidime-avibactam (Li et al., 2021). In this study, *bla*_KPC-190_ was identified as a novel variant arising during ceftazidime-avibactam therapy in a patient with CRKP infection. This variant exhibited resistance to both ceftazidime-avibactam and carbapenems. The reduced hydrolytic activity of KPC-190 towards ceftazidime, coupled with its decreased affinity for avibactam, highlights the complex interplay of structural variations that contribute to its resistance phenotype. This structural characteristic likely makes ceftazidime more susceptible to “capture” by KPC-190 variants compared to the KPC-2 enzyme (Barnes et al., 2017; Alsenani et al., 2022). Ceftazidime contains bulky R1 substituents and small pyridine R2 substituents, which enhance its intrinsic rigidity. This structural feature may influence the two-component regulatory system, thereby contributing to bacterial resistance mechanisms (Alsenani et al., 2022).

The detection rate of KPC variants has risen sharply in recent years, with the number of newly identified *bla*_KPC_ subtypes in the past two years surpassing the total discovered over the previous two decades. These KPC variations are strongly linked to ceftazidime-avibactam treatment (especially in sporadic cases). A recent study reported that up to 48.4% (15/31) of *K. pneumoniae* isolates carrying KPC variants were identified following ceftazidime-avibactam therapy. In our study, the KPC variant developed as a result of prolonged exposure to ceftazidime-avibactam. As the existing studies have shown (Zhang et al., 2023), with the widespread use of ceftazidime-avibactam, the *bla*_KPC_ copy number increases under ceftazidime-avibactam pressure, accompanied by further variations in single *bla*_KPC_.

Unlike the Tn*4401* transposon, the most prevalent *bla*_KPC_-containing mobile element, genomic analysis revealed that *bla*_KPC-190_ is located on an IncFII plasmid within the conserved genetic context IS26-ISKpn8-blaKPC-ΔISKpn6-ΔtnpR- IS26. The presence of two IS26 components is noteworthy, previous studies have shown that many deletions and insertions were accompanied by one or two tandem copies of IS26 (Varani et al., 2021; Shi et al., 2024). This suggests the possibility of IS26-mediated transposition, which may contribute to the diversity of resistance mechanisms and facilitate transmission (Varani et al., 2021).

The evolution of blaKPC-190 presents significant clinical and diagnostic challenges. Conventional detection methods may fail to identify this variant due to enzyme conformational changes, potentially yielding false-negative results. The strain’s atypical susceptibility profile - resistant to ceftazidime-avibactam but variably susceptible to carbapenems - complicates routine antimicrobial susceptibility testing. Chromogenic media (e.g., chromID^®^ CARBA SMART) could support diagnostic surveillance by improving detection of such KPC variants (Bianco et al., 2022). Furthermore, the risk of misidentification as ESBL-producing strains remains a concern, particularly given the current lack of standardized protocols for KPC-variant detection.

Therapeutically, the emergence of KPC-190 highlights the limitations of current treatment regimens for CRKP infections. Although alternative β-lactam/β-lactamase inhibitor combinations, such as meropenem-vaborbactam, show promise, their availability remains limited in many regions. The use of combination therapies, as seen in this case, may delay the onset of resistance but does not prevent the evolution of *bla*_KPC_ variants. Adjusting dosage regimens and incorporating therapeutic drug monitoring (TDM) for ceftazidime-avibactam may mitigate the risk of resistance variations. Additionally, novel diagnostic tools capable of rapidly identifying *bla*_KPC_ variants are essential for guiding treatment decisions.

In conclusion, KPC-190 exemplifies the dynamic evolution of *bla*_KPC_ genes under antibiotic pressure and highlights the urgent need for enhanced global surveillance systems to monitor the emergence of novel KPC variants. Addressing the diagnostic and therapeutic challenges posed by KPC-190 will require a multifaceted approach, including the development of robust diagnostic assays, the optimization of treatment strategies, and the establishment of comprehensive surveillance networks to track the spread of resistant strains. These efforts are critical for mitigating the impact of KPC-190 and preserving the efficacy of current therapeutic options.

## Data Availability

The datasets presented in this study can be found in online repositories. The names of the repository/repositories and accession number(s) can be found in the article/supplementary material.

## References

[B1] Alsenani T. A. Viviani S. L. Kumar V. Taracila M. A. Bethel C. R. Barnes M. D.. (2022). Structural characterization of the D179N and D179Y variants of KPC-2 β-lactamase: Ω-loop destabilization as a mechanism of resistance to ceftazidime-avibactam. Antimicrob Agents Chemother. 66, e0241421. doi: 10.1128/aac.02414-21 35341315 PMC9017313

[B2] Barnes M. D. Winkler M. L. Taracila M. A. Page M. G. Desarbre E. Kreiswirth B. N.. (2017). Klebsiella pneumoniae Carbapenemase-2 (KPC-2), Substitutions at Ambler Position Asp179, and Resistance to Ceftazidime-Avibactam: Unique Antibiotic-Resistant Phenotypes Emerge from β-Lactamase Protein Engineering. mBio 8 (5), e00528-17. doi: 10.1128/mBio.00528-17 29089425 PMC5666153

[B3] Bianco G. Boattini M. Comini S. Leone A. Bondi A. Zaccaria T.. (2022). Implementation of Chromatic Super CAZ/AVI(^®^) medium for active surveillance of ceftazidime-avibactam resistance: preventing the loop from becoming a spiral. Eur. J. Clin. Microbiol Infect. Dis. 41, 1165–1171. doi: 10.1007/s10096-022-04480-x 35933457 PMC9362390

[B4] Boattini M. Bianco G. Bastos P. Comini S. Corcione S. Almeida A.. (2024). Prevalence and mortality of ceftazidime/avibactam-resistant KPC-producing Klebsiella pneumoniae bloodstream infections (2018-2022). Eur. J. Clin. Microbiol Infect. Dis. 43, 155–166. doi: 10.1007/s10096-023-04712-8 37985552 PMC10774640

[B5] Castón J. J. Cano A. Pérez-Camacho I. Aguado J. M. Carratalá J. Ramasco F.. (2022). Impact of ceftazidime/avibactam versus best available therapy on mortality from infections caused by carbapenemase-producing Enterobacterales (CAVICOR study). J. Antimicrob Chemother. 77, 1452–1460. doi: 10.1093/jac/dkac049 35187577

[B6] Chou S. H. Chuang C. Juan C. H. Ho Y. C. Liu S. Y. Chen L.. (2024). Mechanisms and fitness of ceftazidime/avibactam-resistant Klebsiella pneumoniae clinical strains in Taiwan. Int. J. Antimicrob Agents 64, 107244. doi: 10.1016/j.ijantimicag.2024.107244 38925227

[B7] Dietl B. Martínez L. M. Calbo E. Garau J. (2020). Update on the role of ceftazidime-avibactam in the management of carbapenemase-producing Enterobacterales. Future Microbiol 15, 473–484. doi: 10.2217/fmb-2020-0012 32301348

[B8] Findlay J. Bianco G. Boattini M. Nordmann P. (2025). High-level cefiderocol and ceftazidime/avibactam resistance in KPC-producing Klebsiella pneumoniae associated with mutations in KPC and the sensor histidine kinase EnvZ. J. Antimicrob Chemother. 80, 1155–1157. doi: 10.1093/jac/dkaf048 39969122 PMC11962368

[B9] Gonçalves Barbosa L. C. Silva E. S. J. A. Bordoni G. P. Barbosa G. O. Carneiro L. C. (2022). Elevated mortality risk from CRKp associated with comorbidities: systematic review and meta-analysis. Antibiotics (Basel) 11 (7), 874. doi: 10.3390/antibiotics11070874 35884128 PMC9312274

[B10] Hobson C. A. Bonacorsi S. Jacquier H. Choudhury A. Magnan M. Cointe A.. (2020). KPC beta-lactamases are permissive to insertions and deletions conferring substrate spectrum modifications and resistance to ceftazidime-avibactam. Antimicrob Agents Chemother. 64 (12), e01175-20. doi: 10.1128/AAC.01175-20 33020157 PMC7674030

[B11] Huang X. Shen S. Chang F. Liu X. Yue J. Xie N.. (2023). Emergence of KPC-134, a KPC-2 variant associated with ceftazidime-avibactam resistance in a ST11 Klebsiella pneumoniae clinical strain. Microbiol Spectr. 11, e0072523. doi: 10.1128/spectrum.00725-23 37772834 PMC10580995

[B12] Karaiskos I. Daikos G. L. Gkoufa A. Adamis G. Stefos A. Symbardi S.. (2021). Ceftazidime/avibactam in the era of carbapenemase-producing Klebsiella pneumoniae: experience from a national registry study. J. Antimicrob Chemother. 76, 775–783. doi: 10.1093/jac/dkaa503 33249436

[B13] Karakonstantis S. Rousaki M. Kritsotakis E. I. (2022). Cefiderocol: systematic review of mechanisms of resistance, heteroresistance and *in vivo* emergence of resistance. Antibiotics (Basel) 11 (6), 723. doi: 10.3390/antibiotics11060723 35740130 PMC9220290

[B14] Lan P. Lu Y. Jiang Y. Wu X. Yu Y. Zhou J. (2022). Catecholate siderophore receptor CirA impacts cefiderocol susceptibility in Klebsiella pneumoniae. Int. J. Antimicrob Agents 60, 106646. doi: 10.1016/j.ijantimicag.2022.106646 35918032

[B15] Li D. Li K. Dong H. Ren D. Gong D. Jiang F.. (2021). Ceftazidime-Avibactam Resistance in Klebsiella pneumoniae Sequence Type 11 Due to a Mutation in Plasmid-Borne bla (kpc-2) to bla (kpc-33), in Henan, China. Infect. Drug Resist. 14, 1725–1731. doi: 10.2147/IDR.S306095 34007191 PMC8121278

[B16] Li J. Ren J. Wang W. Wang G. Gu G. Wu X.. (2018). Risk factors and clinical outcomes of hypervirulent Klebsiella pneumoniae induced bloodstream infections. Eur. J. Clin. Microbiol Infect. Dis. 37, 679–689. doi: 10.1007/s10096-017-3160-z 29238932

[B17] Li Y. Shen H. Zhu C. Yu Y. (2019). Carbapenem-Resistant Klebsiella pneumoniae Infections among ICU Admission Patients in Central China: Prevalence and Prediction Model. BioMed. Res. Int. 2019, 9767313. doi: 10.1155/2019/9767313 31032370 PMC6457282

[B18] Oliva A. Volpicelli L. Gigante A. Di Nillo M. Trapani S. Viscido A.. (2024). Impact of renal-adjusted ceftazidime/avibactam in patients with KPC-producing Klebsiella pneumoniae bloodstream infection: a retrospective cohort study. JAC Antimicrob Resist. 6, dlae201. doi: 10.1093/jacamr/dlae201 39691790 PMC11649808

[B19] Pemberton O. A. Zhang X. Chen Y. (2017). Molecular basis of substrate recognition and product release by the klebsiella pneumoniae carbapenemase (KPC-2). J. Med. Chem. 60, 3525–3530. doi: 10.1021/acs.jmedchem.7b00158 28388065 PMC5506774

[B20] Poirel L. Walsh T. R. Cuvillier V. Nordmann P. (2011). Multiplex PCR for detection of acquired carbapenemase genes. Diagn. Microbiol Infect. Dis. 70, 119–123. doi: 10.1016/j.diagmicrobio.2010.12.002 21398074

[B21] Sharma R. Park T. E. Moy S. (2016). Ceftazidime-avibactam: A novel cephalosporin/β-lactamase inhibitor combination for the treatment of resistant gram-negative organisms. Clin. Ther. 38, 431–444. doi: 10.1016/j.clinthera.2016.01.018 26948862

[B22] Shi Q. Shen S. Tang C. Ding L. Guo Y. Yang Y.. (2024). Molecular mechanisms responsible KPC-135-mediated resistance to ceftazidime-avibactam in ST11-K47 hypervirulent Klebsiella pneumoniae. Emerg Microbes Infect. 13, 2361007. doi: 10.1080/22221751.2024.2361007 38801099 PMC11172257

[B23] Shi Q. Yin D. Han R. Guo Y. Zheng Y. Wu S.. (2020). Emergence and Recovery of Ceftazidime-avibactam Resistance in blaKPC-33-Harboring Klebsiella pneumoniae Sequence Type 11 Isolates in China. Clin. Infect. Dis. 71, S436–s439. doi: 10.1093/cid/ciaa1521 33367577

[B24] van Duin D. Bonomo R. A. (2016). Ceftazidime/avibactam and ceftolozane/tazobactam: second-generation β-lactam/β-lactamase inhibitor combinations. Clin. Infect. Dis. 63, 234–241. doi: 10.1093/cid/ciw243 27098166 PMC4928383

[B25] Varani A. He S. Siguier P. Ross K. Chandler M. (2021). The IS6 family, a clinically important group of insertion sequences including IS26. Mob DNA 12, 11. doi: 10.1186/s13100-021-00239-x 33757578 PMC7986276

[B26] Wang Y. Wang J. Wang R. Cai Y. (2020). Resistance to ceftazidime-avibactam and underlying mechanisms. J. Glob Antimicrob Resist. 22, 18–27. doi: 10.1016/j.jgar.2019.12.009 31863899

[B27] Winkler M. L. Papp-Wallace K. M. Bonomo R. A. (2015). Activity of ceftazidime/avibactam against isogenic strains of Escherichia coli containing KPC and SHV β-lactamases with single amino acid substitutions in the Ω-loop. J. Antimicrob Chemother. 70, 2279–2286. doi: 10.1093/jac/dkv094 25957381 PMC4500773

[B28] Yang X. Dong N. Chan E. W. Zhang R. Chen S. (2021). Carbapenem resistance-encoding and virulence-encoding conjugative plasmids in klebsiella pneumoniae. Trends Microbiol 29, 65–83. doi: 10.1016/j.tim.2020.04.012 32448764

[B29] Zhang P. Hu H. Shi Q. Sun L. Wu X. Hua X.. (2023). The effect of β-lactam antibiotics on the evolution of ceftazidime/avibactam and cefiderocol resistance in KPC-producing klebsiella pneumoniae. Antimicrob Agents Chemother. 67, e0127922. doi: 10.1128/aac.01279-22 36794957 PMC10019305

